# Direct Growth of van der Waals Tin Diiodide Monolayers

**DOI:** 10.1002/advs.202100009

**Published:** 2021-08-16

**Authors:** Qian‐Qian Yuan, Fawei Zheng, Zhi‐Qiang Shi, Qi‐Yuan Li, Yang‐Yang Lv, Yanbin Chen, Ping Zhang, Shao‐Chun Li

**Affiliations:** ^1^ National Laboratory of Solid State Microstructures and Collaborative Innovation Center of Advanced Microstructures Nanjing University Nanjing 210093 China; ^2^ School of Physics Nanjing University Nanjing 210093 China; ^3^ Key Lab of Advanced Optoelectronic Quantum Architecture and Measurement (MOE) and School of Physics Beijing Institute of Technology Beijing 100081 China; ^4^ Department of Materials Science and Engineering Nanjing University Nanjing 210093 China; ^5^ Institute of Physics and Computational Mathematics Beijing 100088 China; ^6^ Jiangsu Provincial Key Laboratory for Nanotechnology Nanjing University Nanjing 210093 China

**Keywords:** density functional theory, molecular beam epitaxy, scanning tunneling microscopy, SnI_2_, van der Waals monolayers

## Abstract

Two‐dimensional (2D) van der Waals (vdW) materials have garnered considerable attention for their unique properties and potentials in a wide range of fields, which include nano‐electronics/optoelectronics, solar energy, and catalysis. Meanwhile, challenges in the approaches toward achieving high‐performance devices still inspire the search for new 2D vdW materials with precious properties. In this study, via molecular beam epitaxy, for the first time, the vdW SnI_2_ monolayer is successfully fabricated with a new structure. Scanning tunneling microscopy/spectroscopy characterization, as corroborated by the density functional theory calculation, indicates that this SnI_2_ monolayer exhibits a band gap of ≈2.9 eV in the visible purple range, and an indirect‐ to direct‐band gap transition occurs in the SnI_2_ bilayer. This study provides a new semiconducting 2D material that is promising as a building block in future electronics/optoelectronics.

## Graphical abstract

The great application potentials and challenges in high‐performance devices inspire the search for new two‐dimensional (2D) van der Waals (vdW) materials. Via molecular beam epitaxy, the vdW SnI_2_ monolayer is successfully fabricated with a new structure. This SnI_2_ monolayer is a 2D semiconductor with thickness‐dependent properties, which is promising as a building block in future electronics/optoelectronics.

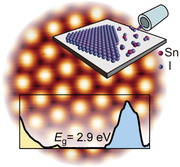

## 1 Introduction

The success of mechanically exfoliated graphene has sparked blowout research interests in two‐dimensional (2D) van der Waals (vdW) materials,^[^ ^1^, ^2^, ^3^, ^4^, ^5^, ^6^, ^7^, ^8^, ^9^, ^10^, ^11^, ^12^, ^13^, ^14^, ^15^, ^16^, ^17^, ^18^, ^19^, ^20^, ^21^, ^22^, ^23^ ^]^ with substantial attention paid to the graphene family,^[^ ^2^, ^3^, ^4^, ^5^, ^6^, ^7^ ^]^ transition metal dichalcogenides (TMDs),^[^ ^8^, ^9^, ^10^ ^]^ 2D metal carbides/nitrides (MXenes),^[^ ^11^, ^12^, ^13^, ^14^, ^15^, ^16^ ^]^ and black phosphorene.^[^ ^17^, ^18^, ^19^, ^20^, ^21^ ^]^ In contrast to the bulk counterparts, these materials at the 2D limit exhibit a diverse range of intriguing properties.^[^ ^24^, ^25^, ^26^, ^27^ ^]^ Yet, challenges still exist in respect of applications for the currently explored 2D materials, such as the gapless band structure of graphene,^[^ ^4^ ^]^ relatively low carrier mobility of TMDs,^[^ ^28^ ^]^ and the poor air stability of black phosphorene.^[^ ^29^, ^30^, ^31^ ^]^ Therefore, it is crucially important to develop new types of 2D layered materials that exhibit new properties complementary to the current 2D material families.

The group IVA metal dihalide has been extensively explored regarding to their superior properties such as the visible‐range band gap and the thickness‐dependent band structure,^[^ ^32^, ^33^, ^34^, ^35^ ^]^ as well as the potential applications in semiconductor optical devices and perovskite solar cells.^[^ ^36^, ^37^, ^38^, ^39^, ^40^, ^41^ ^]^ However, on one hand, the layered 2H‐PbI_2_, as one of the most widely studied materials, is unfriendly to the environment. On the other hand, the bulk SnI_2_ hosts a rather different three‐dimensional (3D) crystal structure,^[^ ^42^ ^]^ and its vdW monolayer has never been experimentally realized.

In this work, for the first time, we successfully grew the vdW monolayers of SnI_2_ via molecular beam epitaxy (MBE) technique. The growth takes a 2D mode, and the thickness is well controllable at the monolayer precision. Scanning tunneling microscopy/spectroscopy (STM/STS) measurements, with the aid of first principle density functional theory (DFT) calculations, confirm that the grown SnI_2_ monolayer hosts a new structure of the layered 2H‐PbI_2_ type. The SnI_2_ monolayer possesses an appreciable indirect band gap of ≈2.9 eV, and an indirect–direct band gap transition occurs as the thickness increases to above two layers. Such transition is opposite to the MoS_2_ monolayers that exhibit a direct‐indirect band gap transition as the thickness increases.^[^ ^8^ ^]^

## 2 Results

**Figure 1:**
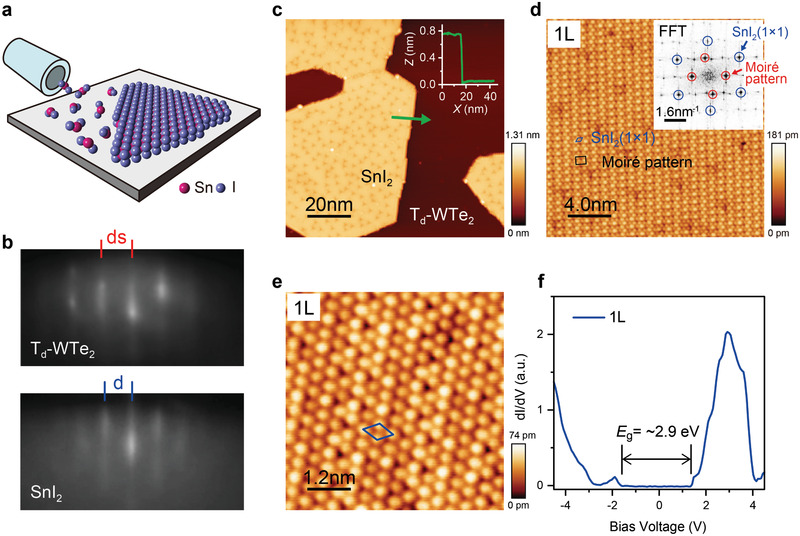
Epitaxial growth, structural, and electronic characterizations of vdW SnI_2_ monolayers grown on Td‐WTe_2_. a) Schematic illustration of the growth of hexagonal SnI_2_ monolayer on Td‐WTe_2_. b) RHEED patterns of epitaxial SnI_2_ film and Td‐WTe_2_ substrate. c) Large‐scale STM image of SnI_2_ monolayer (size: 100 × 100 nm^2^, *U* = +2 V, *I*_t_ = 100 pA). Inset: height profile of line scan across the step indicated by the green arrowed line. d) High‐resolution STM image taken on the SnI_2_ monolayer (size: 20 × 20 nm^2^, *U* = −700 mV, *I*_t_ = 100 pA). The surface unit cells of SnI_2_ monolayer and the Moiré pattern are marked in blue and black, respectively. Inset: the corresponding FFT pattern with the blue and red circles marked the reciprocal lattice peaks of SnI_2_‐(1 × 1) and the Moiré pattern respectively. e) Atomic resolution of monolayer SnI_2_. The blue rhombus represents the unit cell (size: 6 × 6 nm^2^, *U* = −1 V, *I*_t_ = 100 pA). It indicates the hexagonal lattice of a = b = ≈4.48 Å. f) Typical d*I*/d*V* spectra taken on monolayer SnI_2_ (*U* = +2.2 V, *I*_t_ = 100 pA, modulation: 40 mV). The black lines indicate the band edges.

The growth procedure of the epitaxial SnI_2_ monolayers on orthorhombic Td‐phase WTe_2_ (Td‐WTe_2_) is illustrated in **Figure** **1**a. We emphasize that the high‐quality SnI_2_ monolayer can be directly grown on the substrate kept at room temperature (≈20–22 °C), without further annealing. The reflection high‐energy electron diffraction (RHEED) pattern was in situ measured to monitor the morphology of SnI_2_ monolayers. As displayed in Figure 1b, the streaks in the RHEED pattern indicate the 2D growth mode of SnI_2_ on the Td‐WTe_2_ substrate. Quantitatively, the streak spacing for the SnI_2_ film (*d*) is ≈1.27 times that of the Td‐WTe_2_ substrate (ds). The surface morphology of ≈0.5 monolayer (ML) SnI_2_ deposited on the Td‐WTe_2_ substrate (Figure 1c) verifies the formation of atomically flat and hexagonal‐shaped SnI_2_ islands. The step height of the SnI_2_ islands, as obtained through the line‐scan profile (inset to Figure 1c) is ≈0.7 nm high. Atomically resolved STM images (Figure 1d,e), together with the corresponding fast Fourier transformation (FFT), clearly evidence the hexagonal lattice symmetry. The lattice parameter is determined to be *a* = *b* = ≈4.48 Å, in accordance with the quantitative analysis of the RHEED data. Besides, the FFT result (inset to Figure 1d) also exhibits extra dots, which originated from the Moiré pattern formed between the SnI_2_ monolayer and Td‐WTe_2_ substrate, and can also be clearly observed in the STM image in Figure 1d (marked in black). It is noteworthy that the grown SnI_2_ monolayer is in a new structural phase, which is completely different from its bulk counterpart. As previously reported,^[^ ^42^ ^]^ the bulk SnI_2_ crystallizes in the 3D monoclinic structure with the space group of *C*2/*m*.

Randomly distributed defects are observed on the SnI_2_ islands, particularly on the SnI_2_ monolayer (Figure 1c). More STM data indicate that three different defects can be identified, among which the type A is the majority, and types B and C are occasionally observed (see Figure S5, Supporting Information). In combination with DFT simulation, we ascribe the type A defect as the extra I atom at the bottom of SnI_2_ monolayer, and types B and C as the extra Sn atom at the bottom and I vacancy on the top, respectively.

Figure S4a, Supporting Information, shows the typical large‐scale STM image of SnI_2_ full monolayer, which indicates the existence of multiple domains. The average size of SnI_2_ single domain is about 100–150 nm. We further took the atomically resolved STM image between two adjacent domains to elucidate the boundary structure (Figure S4b,c, Supporting Information). Even though the two adjacent domains exhibit different lattice orientations, the growth at the boundary is seamless.

To further explore the electronic structure of the SnI_2_ monolayer, differential conductance (d*I*/d*V*) spectra, which reflect the local density of states, were measured. Figure 1f presents the typical d*I*/d*V* spectrum taken on the SnI_2_ monolayer away from defects (more data can be found in Figure S1, Supporting Information). The semiconducting nature of the SnI_2_ monolayer is revealed, and a wide band gap of ≈2.9 eV is determined, within the range of visual purple spectrum. The valence band maximum (VBM) and conduction band minimum (CBM) are also identified at ≈−1.6 eV (below Fermi energy) and ≈+1.3 eV (above Fermi energy).

**Figure 2:**
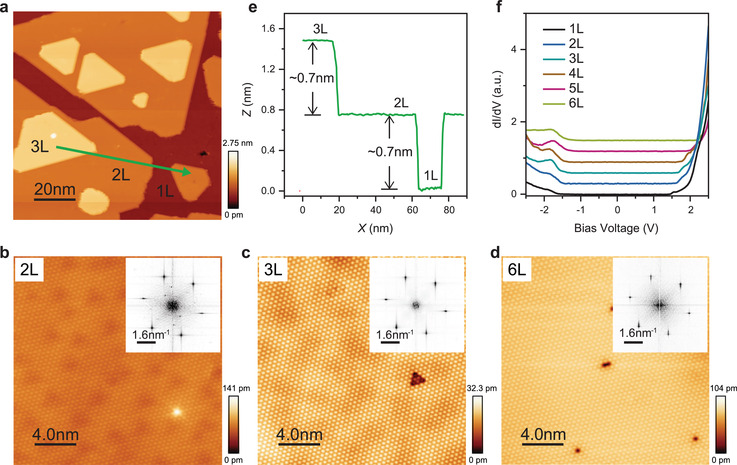
Van der Waals growth of vdW multilayer of SnI_2_ on Td‐WTe_2_. a) Large‐scale STM topographic image of multilayered SnI_2_ films with thickness varying from 1L to 3L (size: 100 × 100 nm^2^, *U* = −2 V, *I*_t_ = 100 pA). b–d) Atomic resolutions (20 × 20 nm^2^) obtained on 2L, 3L, and 6L, respectively (*U* = −1.5 V, *I*_t_ = 50 pA). Inset: the corresponding FFT images. e) Line scan profile across from 3L to 1L along the green allowed line in (a). f) d*I*/d*V* spectra taken on SnI_2_ with different thicknesses ranging from 1L to 6L.

Multilayered films can also be epitaxially grown with a monolayer precision. **Figure** **2**a shows the surface of the multilayered SnI_2_ films with thicknesses varied from monolayer (1L) to three layers (3L). Atomically resolved STM images, Figure 2b–d, demonstrate that the layered hexagonal lattices are maintained in these SnI_2_ multilayers, and no structural transition to the bulk phase is observed (more data can be found in Figure S2, Supporting Information). It is noteworthy that the defect concentrations in SnI_2_ bilayer or multilayers, typically the extra I atoms at the bottom, are significantly lower than the monolayer, which can be attributed to the different adsorption and diffusion of I atoms at the SnI_2_ interlayer from SnI_2_/WTe_2_ interface. We further calculated the formation energies of the extra iodine defect at the SnI_2_/WTe_2_ and SnI_2_/SnI_2_ interfaces. Our calculation results indicate that the extra iodine defect formed at the SnI_2_/WTe_2_ interface is more stable than at the SnI_2_/SnI_2_ interface by ≈0.3 eV. Interestingly, the interlayer spacing for the multilayered SnI_2_ (Figure 2e) keeps a near constant of ≈0.7 nm, the same as the step height for the monolayer, indicating no identifiable layer‐dependent interlayer coupling. All of these results collectively suggest that such hexagonal vdW structure can be stabilized in the 2D limit, although it does not exist in the bulk.

The d*I*/d*V* spectra (Figure 2f) taken on the SnI_2_ monolayers indicate that the width of gap keeps nearly the same for the thickness up to six layers (6 L) (The spatial uniformity of the STS spectra are verified by the STS line scans, as shown in Figure S1, Supporting Information). This differs from most of the other TMD vdWs materials hosting the electronic structures sensitive to the number of layers.^[^ ^24^, ^43^, ^44^, ^45^, ^46^, ^47^, ^48^, ^49^, ^50^ ^]^ The locations of CBM and VBM can be extracted from the d*I*/d*V* data in the logarithmic form (see Figure S6b, Supporting Information). As the thickness is decreased to monolayer, the Fermi energy (*E*_F_) gradually shifts upwards with respect to the band edges, which may be due to the electron doping effect from the intrinsic defects or the substrate. Such doping effect becomes prominent in the proximity of interface, similar to what was reported for GaSe/graphene heterostructures.^[^ ^50^ ^]^

**Figure 3:**
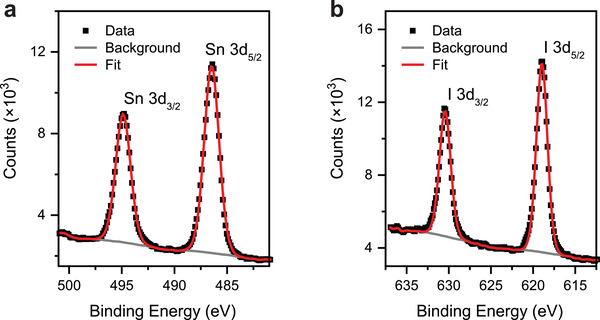
XPS results of the as‐grown vdW SnI_2_ monolayers. a) Sn 3d and b) I 3d XPS spectra of SnI_2_ films.

To investigate the chemical stoichiometry of the grown SnI_2_ monolayers, X‐ray photoelectron spectroscopy (XPS) measurements were ex Situ performed after exposure in air. **Figure** **3**a,b displays the XPS spectra of Sn 3d and I 3d core levels, respectively. The binding energy of Sn 3d5/2 (486.4 eV), Sn 3d3/2 (494.8 eV), I 3d5/2 (619 eV), and I3d3/2 (630 eV) reflects the valence states of Sn (+2) and I (−1) for the SnI_2_ compound.^[^ ^51^ ^]^ The chemical stoichiometry for Sn:I is quantitatively determined to be ≈1:2, consistent with the ideal SnI_2_ compound. Moreover, there is no oxidation state of SnI_2_ detected within the instrumental resolution. Thus, it is concluded that the as‐grown SnI_2_ monolayers is significantly inert to air.

**Figure 4:**
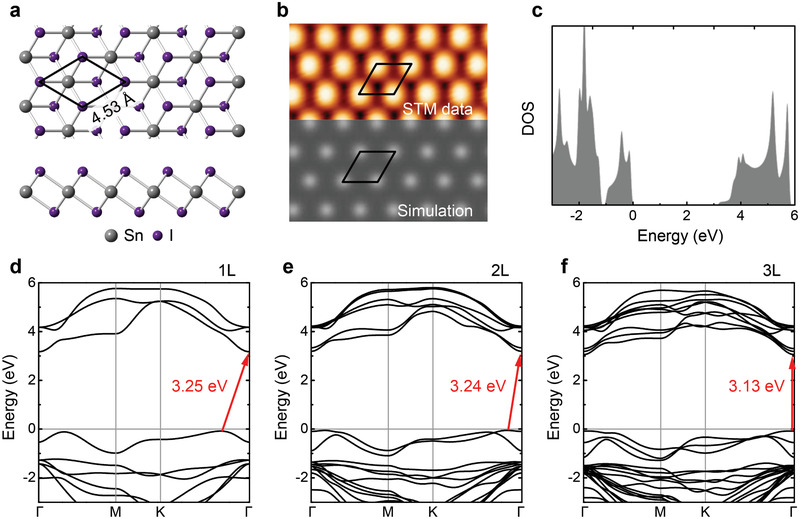
Atomic structure of vdW SnI_2_ monolayers and the indirect‐ to direct‐transition of band gap. a) Top and side views of the atomic structure of SnI_2_. b) Experimental and simulated STM images of SnI_2_ monolayer. c) DOS of SnI_2_ monolayer calculated via the GW method. d–f) Calculated GW band structures of 1L, 2L, and 3L, respectively. The red arrows indicate the lowest energy transitions.

The DFT optimized atomic models of the SnI_2_ mono‐ and multi‐layers are constructed. The monolayer structure is shown in **Figure** **4**a. It adopts the 2H‐PdI_2_ type (space group: *P*3*m*1) structure. Each monolayer comprises three atomic planes covalently bonded in the sequence of I‐Sn‐I with the in‐plane lattice of *a* = *b* = 4.53 Å. In multilayers, the separation between adjacent layers governed by vdW interactions is ≈0.32 nm. Both theoretical atomic structures and lattice constants agree well with the experimental results. Moreover, the calculated band structure indicates that monolayer SnI_2_ has an indirect semiconducting gap of ≈3.25 eV, as shown in Figure 4d. Even though there still exists a minor discrepancy between the calculated and experimentally observed gap value (≈2.9 eV), the DFT + GW calculation method we applied corrects the usual underestimation of energy gap by DFT method. The corresponding calculated DOS based on the proposed crystal structure (Figure 4c) agrees well with the experimental d*I*/d*V* spectra, further demonstrating the as‐grown SnI_2_ monolayer to be a new 2D layered semiconductor. The calculated band structure of SnI_2_ (Figure 4d–f) shows that the monolayer and bilayer host the indirect‐gap, and a transition to direct‐gap occurs on the trilayer SnI_2_, opposite to hexagonal 2H‐phase MoS_2_ (2H‐MoS_2_) whose direct band gap only exists in monolayer.^[^ ^43^ ^]^ We also considered the other possible stacking, the common structure for semiconducting TMDs, such as MoS_2_. However, the calculated energy is significantly higher, and the energy difference is ≈0.56 eV per unit cell. Please note that a nomenclature inconsistency exists between 2H‐PbI_2_ and TMDs. In fact, the crystal structure of 2H‐PbI_2_ corresponds to the 1*T* structure, as referred to in TMD compounds, which is different from the common structure of 2H‐MoS_2_.

This substrate‐assisted growth of the 2D hexagonal SnI_2_ monolayer is universal, regardless of the lattice symmetry and lattice constants of the substrate. As shown in Figure S3, Supporting Information, the SnI_2_ monolayer with the hexagonal lattice can also be successfully grown on the bilayer graphene (BLG)/SiC substrates at room temperature (≈20–22 °C). The SnI_2_ monolayer grown on the graphene/SiC substrate exhibits an irregular shape, which differs from the equilateral triangular SnI_2_ grown on the Td‐WTe_2_ substrate. This is probably due to the pinning effect triggered by the native defects on the graphene/SiC surface (see Figure S7, Supporting Information). We also find that both its structural and electronic properties are similar to the SnI_2_ monolayers on the Td‐WTe_2_ substrate. Particularly, their similar properties include the atomic structure and in‐/out‐plane lattice parameters, 2D vdW growth mode as manifested by the streaky RHEED patterns, air stability as suggested by the XPS spectra, and semiconducting nature with a comparable energy gap of ≈2.94 eV. In view of the distinct atomic structures of hexagonal graphene and orthorhombic Td‐WTe_2_, these results show that the growth of semiconducting SnI_2_ with layered hexagonal lattice is barely influenced by the different lattice symmetry of substrates.

## 3 Discussions

The bulk SnI_2_ is a monoclinic structure with *a* = 14.17 Å, *b* = 4.535 Å, and *c* = 10.87 Å (space group: *C*2/*m*).^[^ ^42^ ^]^ The DFT calculated free energy per unit volume of the bulk monoclinic SnI_2_ is ≈6.7 meV lower than that of the layered hexagonal SnI_2_, suggesting that the layered phase is less stable. However, in the 2D limit, the formation of monolayer or few layers of the bulk phase requires the interlayer bonds of monoclinic SnI_2_ to be broken; consequently, the energy needed will be higher than that in the 2D hexagonal phase. Therefore, the hexagonal phase of SnI_2_ in the monolayer limit is more stable, which is consistent with our experimental results. We also addressed the kinetic issue for the growth of 2D hexagonal phase. We calculated the adsorption energies of the SnI_2_ layer on the WTe_2_ substrate and another SnI_2_ layer, respectively. The calculation results show that the adsorption energy of the SnI_2_ on WTe_2_ is higher than that on another SnI_2_ layer by ≈75 meV per unit cell. Therefore, the SnI_2_ prefers to adsorb on the WTe_2_ substrate, instead of another SnI_2_ layer, which favors the 2D growth. The hexagonal monolayer becomes kinetically trapped in the 2D limit, owing to the lower surface energy.

## 4 Conclusion

In summary, we have successfully fabricated vdW SnI_2_ monolayers. The substrates with different lattice symmetries, such as the orthorhombic Td‐WTe_2_ and hexagonal graphene, are both feasible to the epitaxy of vdW SnI_2_ monolayer. XPS data manifested its stability in air. The grown SnI_2_ monolayer is found to be a new layered vdW semiconductor with an appreciable band gap of ≈2.9 eV. The SnI_2_ monolayer also exhibits thickness‐dependent properties, for example, the indirect‐ to direct‐band gap transition. The experimental realization of SnI_2_ monolayers, a new 2D semiconductor with thickness‐dependent properties, provides an optimal material candidate for applications in electronics and optoelectronics.

## 5 Experimental Section

### MBE Growth of SnI_2_ Films

The SnI_2_ monolayers were grown on the Td‐WTe_2_ and BLG/SiC substrates using the MBE. The Td‐WTe_2_ substrates were obtained via in situ cleaving the Td‐WTe_2_ single crystal in ultrahigh vacuum (UHV), and the BLG/SiC substrates were obtained by repeatedly flashing the SiC single crystal for more than three times in UHV with a base pressure lower than 1 × 10^−10^ Torr. Prior to the SnI_2_ growth, the surface qualities of Td‐WTe_2_ and BLG/SiC substrates were checked via STM characterization. Anhydrous SnI_2_ powder (Alfa Aesar, 99.999%) was loaded in a Knudsen diffusion cells as the evaporation source. The growth morphology was in situ monitored by RHEED. During the growth, the SnI_2_ source was heated to ≈280 °C, and the substrates were kept at room temperature (≈20–22 °C).

### Scanning Tunneling Microscopy Characterization

The STM measurements were in situ carried out with a commercial low‐temperature UHV‐STM system (Unisoku, USM1500). The base pressure was lower than 1 × 10^−11^ Torr. A mechanically polished Pt‐Ir tip was used for scanning under the constant‐current mode. The d*I*/d*V* spectra were collected using the lock‐in amplifying technique with an AC modulation of ≈10 mV at a frequency of 879 Hz.

### Density Functional Theory Calculation

The DFT calculations in this work were performed using the projected augmented wave method,^[^ ^52^ ^]^ as implemented in the Vienna ab initio simulation package.^[^ ^53^ ^]^ The exchange correlation potential was described by the generalized gradient approximation of Perdew–Burke–Ernzerhof type.^[^ ^54^ ^]^ An energy cutoff of 250 eV was used, which was converged in the authors' test. The Brillouin zone was sampled by a 16 × 16 × 1 k‐point mesh. To accurately describe the interlayer interactions, the D2 vdW correction method presented by Grimme was adopted.^[^ ^55^ ^]^ In all the slab models, the vacuum distances were larger than 15 Å. This distance was large enough to avoid the interaction between two nearest slabs. The atomic structures were carefully relaxed until the forces on each atom were less than 0.01 eV Å^−1^. To get accurate electronic structures, the authors also performed the GW calculations in the G0W0 level.^[^ ^56^ ^]^

## Conflict of Interest

The authors declare no conflict of interest.

## Author Contributions

Q.‐Q.Y. and F.Z. contributed equally to this work. S.‐C.L. conceived the project. Q.‐Q.Y. grew the SnI_2_ monolayers and carried out STM experiments with the assistance of Z.‐Q.S. and Q.‐Y.L. F.Z. and P.Z. carried out the DFT and GW theoretical calculations. Y.‐Y.L. and Y.C. grew the single‐crystal WTe_2_ substrates. Q.‐Q.Y. and S.‐C.L. wrote the manuscript with the input from F.Z and P.Z. All authors discussed the results and commented on the manuscript.

## Supporting information

Supporting Information

## Data Availability

The data that support the findings of this study are available from the corresponding author upon reasonable request.
