# Supplementary material for: Direct Growth of van der Waals Tin Diiodide Monolayers
Source: Adv Sci (Weinh). 2021 Aug 16;8(20):2100009. doi: 10.1002/advs.202100009 (PMC8529434; doi:10.1002/advs.202100009)
Supplement: Supplementary file 1 — Supporting Information [file ADVS-8-2100009-s001.pdf]

© 2021 Wiley-VCH GmbH

ADVANCED SCIENCE
Open Access

Supporting Information

for *Adv. Sci.*, DOI: 10.1002/advs.202100009

Direct Growth of van der Waals Tin Diiodide Monolayers

Qian-Qian Yuan, Fawei Zheng, Zhi-Qiang Shi, Qi-Yuan Li, Yang-Yang Lv, Yanbin Chen, Ping Zhang, and Shao-Chun Li**

Supporting Information

Direct growth of van der Waals tin diiodide monolayers

Qian-Qian Yuan[†], *Fawei Zheng*[†], *Zhi-Qiang Shi, Qi-Yuan Li, Yang-Yang Lv, Yanbin Chen, Ping Zhang*[*], *and Shao-Chun Li*[*]

Dr. Q.-Q. Y.[†], Dr. Z.-Q. S., Q.-Y. L., Dr. Y.-Y. L., Prof. Y. C., Prof. S.-C. L.[*]
National Laboratory of Solid State Microstructures and Collaborative Innovation Center of Advanced Microstructures,
Nanjing University,
Nanjing 210093, China
E-mail: scli@nju.edu.cn

Dr. Q.-Q. Y.[†], Dr. Z.-Q. S., Q.-Y. L., Prof. Y. C., Prof. S.-C. L.[*]
School of Physics,
Nanjing University,
Nanjing 210093, China

Prof. F. Z.[†]
Key Lab of Advanced Optoelectronic Quantum Architecture and Measurement (MOE) and School of Physics,
Beijing Institute of Technology,
Beijing 100081, China

Dr. Y.-Y. L.
Department of Materials Science and Engineering,
Nanjing University,
Nanjing 210093, China

Prof. P. Z.[*]
Institute of Physics and Computational Mathematics,
Beijing 100088, China
E-mail: zhang_ping@iapcm.ac.cn

Prof. S. -C. L.[*]
Jiangsu Provincial Key Laboratory for Nanotechnology,
Nanjing University,
Nanjing 210093, China

The uniformity of the dI/dV spectra on the SnI_2 films.

As shown in Figure S1a and b, even though there exists a certain number of defects on the monolayer SnI_2, the spatially resolved differential conductance (d*I*/d*V*) spectra display the uniformity of electronic structure with constant band gap size. There is no shift of the main features exist but only a change of the intensity. Similar to the monolayer, the d*I*/d*V* spectra taken on 2L and 3L surfaces also show the uniformity (Figure S1c-f).

The successful growth of SnI_2 monolayers on graphene/SiC substrates.

To test the effect of the substrates on the epitaxial SnI_2 monolayers, i.e., the lattice, the graphene/SiC with hexagonal lattice was chosen for comparing with Td-WTe_2 as discussed in the main text. As shown in Figure S3d, an atomically flat substrate of graphene/SiC was obtained by being repeatedly flashed. Through the similar growth conditions for SnI_2 monolayers on Td-WTe_2, including temperature of substrate and sources, we successfully fabricated the SnI_2 monolayers on graphene/SiC substrates (Figure S3a). The RHEED study shown in Figure S3b, combined with the atomically resolved STM image shown in Figure S3e illustrate the hexagonal lattice of a= 4.5 Å, which is comparable to that of SnI_2 monolayer grown on Td-WTe_2. The STS measurements are performed to investigate the electronic properties. As displayed in Figure S3f, a band gap of approximately 2.9 eV was recognized, revealing the semiconducting nature. Additionally, no oxidation exists in the ex-suit XPS spectra displayed in Figure S3c, which demonstrates its air-stability.

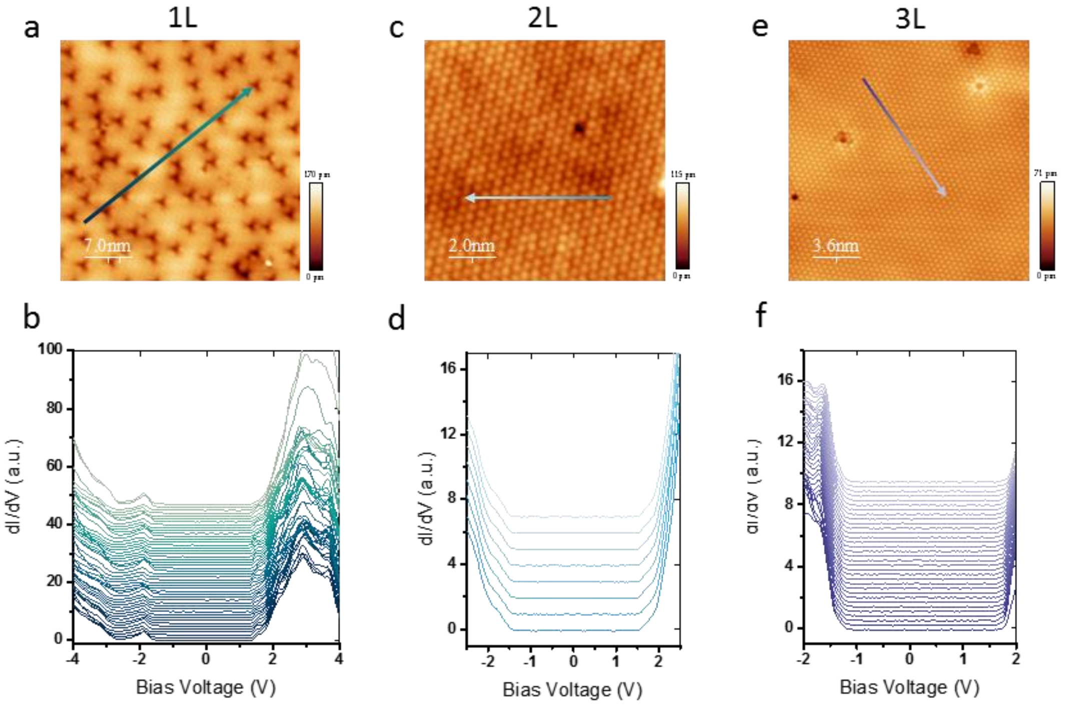

Figure S1. Spatially resolved dI/dV spectra taken on different-thickness SnI_2 films. (a) STM topographic image taken on the surface of monolayer SnI_2 (size: 35 × 35 nm^2, U= +2 V, I_t= 100 pA). (c) STM topographic image taken on 2L SnI_2 (10 × 10 nm^2, U= -2 V, I_t= 50 pA). (e) STM topographic image taken on 3L SnI_2 (18 × 18 nm^2, U= -2 V, I_t= 50 pA). The arrowed lines indicate the positions for the STS measurements. (b, d, f) dI/dV spectra taken on the positions as marked by the same colored arrowed lines in (a, c, e) respectively. All the dI/dV spectra were taken on the defect-free regions.

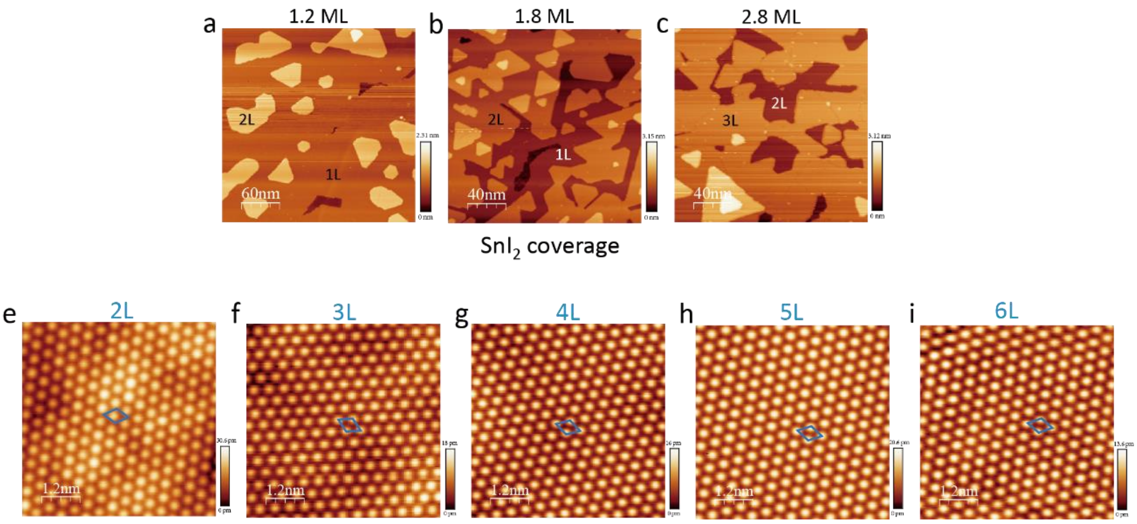

Figure S2. Characterization of epitaxial multilayered SnI_2 grown on WTe_2. (a-c) Large-scale STM images of SnI_2 films with coverages of 1.2 ML, 1.8 ML and 2.8 ML respectively (size: 200 × 200 nm^2, U= -2 V, I_t= 100 pA). (e-i) The atomically-resolved STM images taken on 2L to 6L (size: 6 × 6 nm^2). They are taken at U= -1.5 V, I_t= 50 pA.

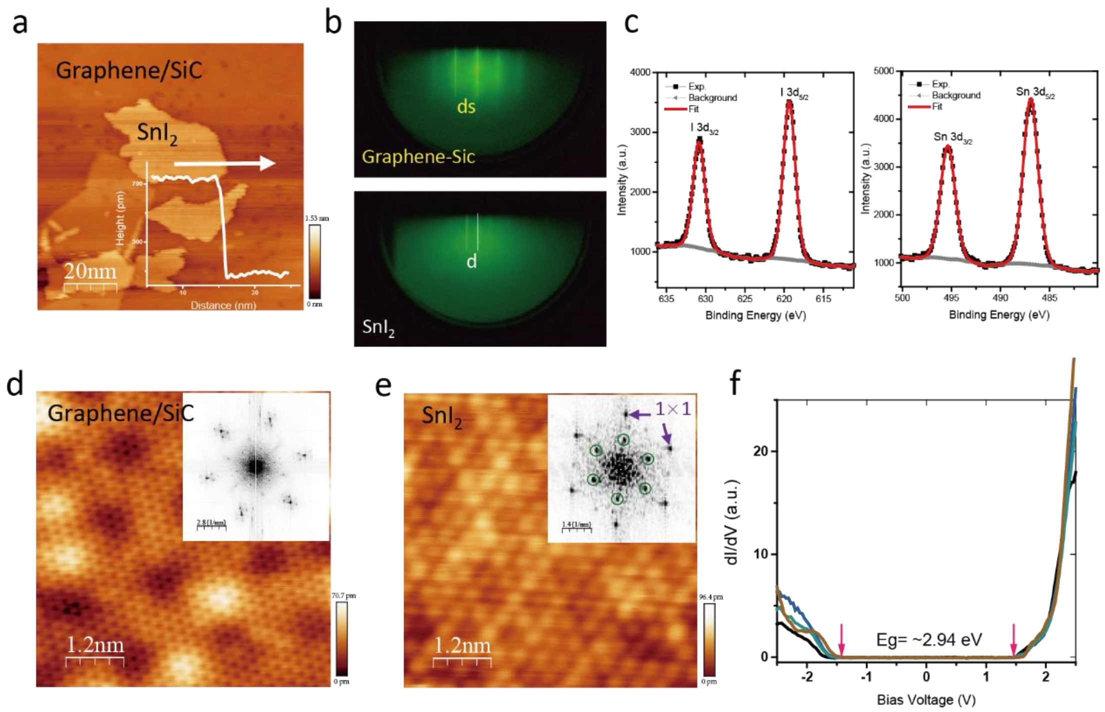

Figure S3. The characterization of structural and electronic properties of SnI_2 monolayer grown on graphene/SiC. (a) STM topographic image of SnI_2 monolayer (size: 100 × 100 nm^2, U= +4 V, I_t= 50 pA). Inset: line-scan height profile along the white arrowed line. (b) RHEED patterns obtained on the grown SnI_2 films and the graphene/SiC substrate. (c) XPS spectra of Sn 3d and I 3d electrons. (d) High-resolved STM image of graphene/SiC substrate (size: 6 × 6 nm^2, U= +500 mV, I_t= 50 pA). Inset: the corresponding FFT image. (e) Atomically resolved image taken on the grown SnI_2 monolayer (size: 6 × 6 nm^2, U= +1.5 V, I_t= 50 pA). Inset: the corresponding FFT image where the peaks indicated by purple arrows reflect the 1x1 surface and the peaks in green circles reflect the Morrie pattern resulted from the mismatch of lattices between epitaxial SnI_2 and graphene/SiC substrate. (f) The dI/dV spectra taken on the SnI_2 surfaces. They were taken at U= +2 V, I_t= 50 pA and the applied modulation is 15 mV. The red arrows mark the band gap edges.

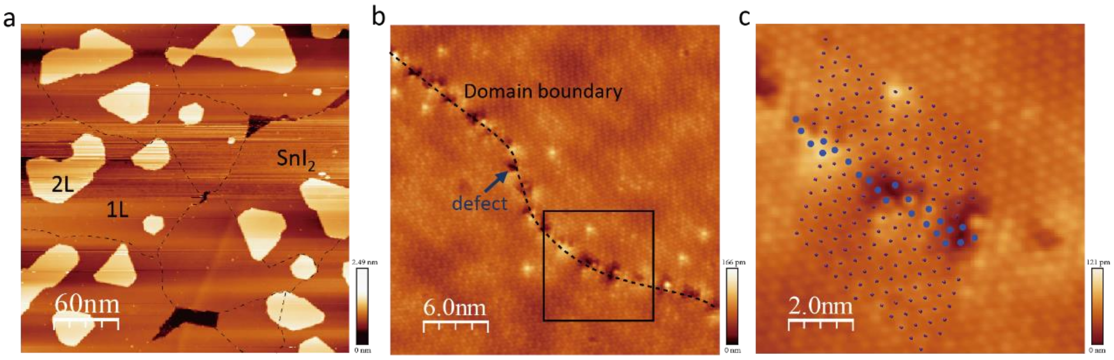

Figure S4. STM characterization of the SnI_2 domain boundary. (a) Large-scale STM image taken on the SnI_2 full monolayer surface (size: 300 × 300 nm^2, U= -2 V, I_t= 100 pA). The black dashed lines mark the domain boundaries. (b) High-resolution STM image of a domain boundary (size: 30 × 30 nm^2, U= -2 V, I_t= 100 pA). The black dashed line marks the boundary. The blue arrow indicates the defect. (c) The zoom-in image form the black square region in (b). The atomic arrangement at the two domains are marked by small blue balls and that at the boundary is marked by bigger blue balls.

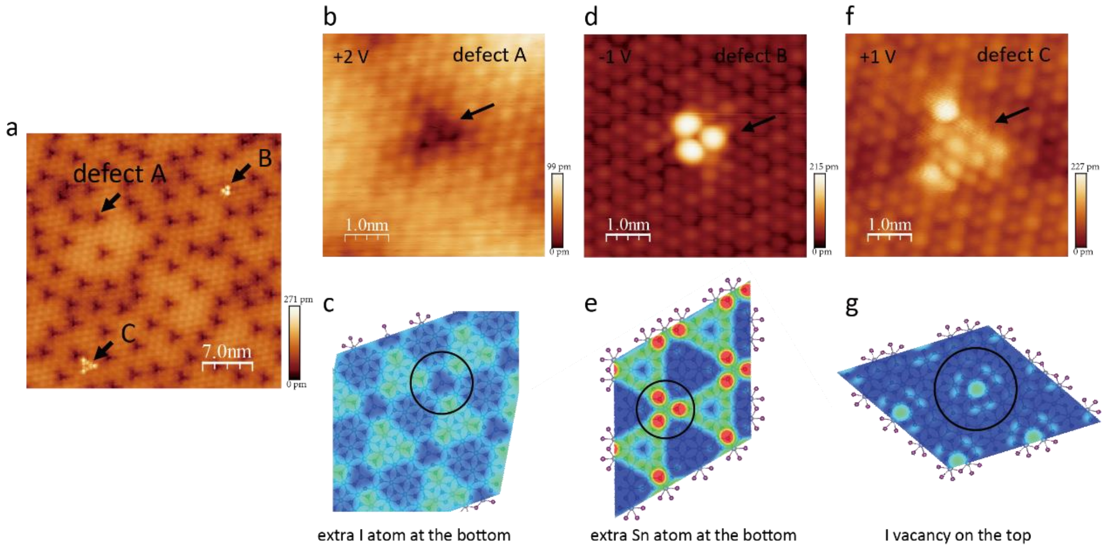

Figure S5. Randomly distributed defects on SnI_2 monolayer. (a) Large-scale STM image taken on SnI_2 monolayer (size: 35 × 35 nm^2, U= +1.8 V, I_t= 100 pA). (b, d, f) Atomically resolved STM images of defect A, defect B and defect C respectively (size: 5 × 5 nm^2). They were taken at U= +2 V (I_t= 100 pA), -1 V (I_t= 100 pA) and +1 V (I_t= 100 pA) respectively. (c, e, g) DFT simulated STM images of the surfaces with one extra I atom at the surface bottom, one extra Sn atom at the surface bottom and one I vacancy on the surface top respectively. The color ranges from blue to red represent the height from low to high. The black circles in the STM simulations mark the locations of the defects.

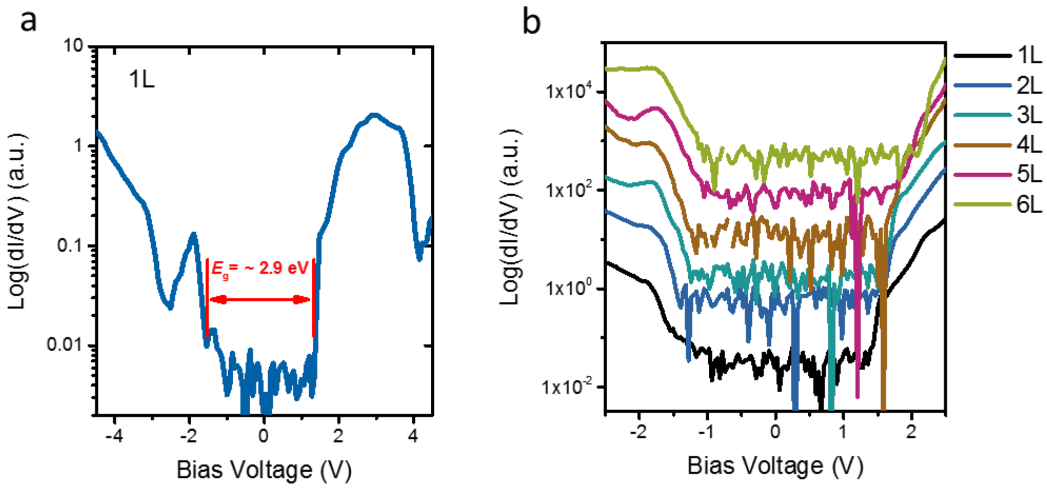

Figure S6. The logarithmic form of the dI/dV spectra shown in Figure 1f and Figure 2f in the manuscript.

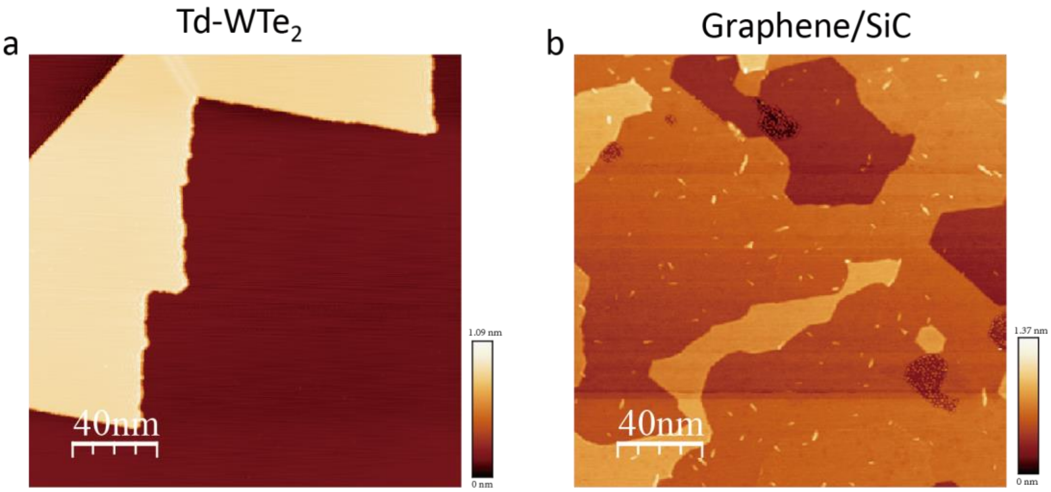

Figure S7. STM characterizations of the substrates prior to the SnI_2 growth. Large-scale STM images of Td-WTe_2 substrate (a) and graphene/SiC substrate (b). They were both taken at U= +1.0 V, I_t= 100 pA.
